# Insulin resistance and central obesity determine hepatic steatosis and explain cardiovascular risk in steatotic liver disease

**DOI:** 10.3389/fendo.2023.1244405

**Published:** 2023-09-29

**Authors:** Georg Semmler, Lorenz Balcar, Sarah Wernly, Andreas Völkerer, Lorenz Semmler, Laurenz Hauptmann, Bernhard Wernly, Elmar Aigner, David Niederseer, Christian Datz

**Affiliations:** ^1^ Division of Gastroenterology and Hepatology, Department of Internal Medicine III, Medical University of Vienna, Vienna, Austria; ^2^ Department of Internal Medicine, General Hospital Oberndorf, Teaching Hospital of the Paracelsus Medical University Salzburg, Salzburg, Austria; ^3^ Department of Plastic, Reconstructive and Aesthetic Surgery, Medical University of Vienna, Vienna, Austria; ^4^ First Department of Medicine, Paracelsus Medical University Salzburg, Salzburg, Austria; ^5^ Department of Cardiology, Hochgebirgsklinik Davos, Davos, Switzerland; ^6^ Christine Kühne Center for Allergy Research and Education (CK-CARE), Davos, Switzerland; ^7^ Department of Cardiology, University Hospital Zurich, University Heart Center, University of Zurich, Zurich, Switzerland

**Keywords:** MASLD, NAFLD, steatotic liver disease, metabolic syndrome, insulin resistance, SCORE2

## Abstract

**Background:**

Metabolic dysfunction-associated steatotic liver disease (MASLD) has recently been proposed to replace non-alcoholic fatty liver disease and focus on patients with progressive disease due to the presence of metabolic dysfunction. However, it is unclear whether the new definition actually identifies patients with hepatic steatosis at increased cardiovascular risk.

**Methods:**

A total of 4,286 asymptomatic subjects from the SAKKOPI study aged 45–80 years undergoing screening colonoscopy were analyzed. Steatosis was diagnosed by abdominal ultrasound. MASLD was diagnosed according to the recent expert consensus. Insulin resistance was assessed by homeostasis model assessment-insulin resistance score (HOMA-IR) (cutoff: ≥2.5), subclinical inflammation was estimated by ferritin/CRP/uric acid, and cardiovascular risk was assessed using SCORE2/ASCVD.

**Results:**

Mean age was 59.4 ± 8.5 years, 51.6% were male; mean BMI was 27.0 ± 4.5 kg/m², 9.2% had type 2 diabetes mellitus. In total, 1,903 (44.4%) were diagnosed with hepatic steatosis and were characterized by more severe metabolic dysfunction including insulin resistance (47.1% vs. 12.2%, *p* < 0.001) and central obesity (waist circumference ≥102/88 cm, 71.8% vs. 37.1%, *p* < 0.001). This translated into higher (subclinical) inflammation (ferritin 153 vs. 95 mg/dL, *p* < 0.001, uric acid 6.3 mg/dL vs. 5.2 mg/dL, *p* < 0.001) and 10-year cardiovascular risk (SCORE2 7.8 points vs. 5.1 points, *p* < 0.001, ASCVD 17.9 points vs. 10.8 points, *p* < 0.001). 99.0% of subjects with steatosis met the MASLD definition, 95.4% met the MAFLD definition, and 53.6% met the definition of metabolic syndrome, while 95.4% of subjects without steatosis also met the MASLD criteria for metabolic dysfunction compared to 69.0% and 17.4% who met the MAFLD and metabolic syndrome criteria, respectively. Forward stepwise regression indicated that waist circumference, HOMA-IR, and triglycerides were most relevant in explaining the presence of hepatic steatosis across all subgroups of increasing metabolic dysfunction. At the same time, hepatic steatosis was not associated with cardiovascular risk in the overall cohort (SCORE2: *B* = 0.060, 95% CI: −0.193–0.314, and *p* = 0.642) and in patients with metabolic dysfunction after adjusting for age, sex, and these three metabolic dysfunction components.

**Conclusion:**

Although hepatic steatosis is associated with increased central obesity and insulin resistance, metabolic dysfunction *per se* rather than hepatic steatosis explains cardiovascular risk in these patients.

## 1 Introduction

Steatotic liver disease (SLD) is the most prevalent, yet fastest emerging liver disease of the 21st century. In 2020, the hepatology community introduced the novel term “metabolic dysfunction-associated fatty liver disease” (MAFLD) to replace “non-alcoholic fatty liver disease” (NAFLD) (1). Recently, this term was again replaced by “metabolic dysfunction-associated steatotic liver disease” (MASLD), along with a revised definition of metabolic dysfunction (2). With the aim of reducing stigma and being inclusive, it is currently unclear whether this approach effectively identifies the patient population at higher risk, particularly with regard to cardiovascular disease (CVD). Although NAFLD is considered to be a risk factor for CVD (3–5), it is still controversial whether steatosis *per se* promotes this association, or whether other unmeasured factors drive this relationship (6, 7), especially since population-based studies or large-scale meta-analyses often lack complete adjustment for confounders.

Recently, several studies have highlighted the importance of insulin resistance and waist circumference in the development of hepatic steatosis (8, 9). In particular, insulin resistance seems to be the most important driver for hepatic inflammation leading to fibrosis in addition to genetic factors (9, 10). However, these two factors are rarely adjusted for when reporting on cardiovascular outcomes. Thus, we set out to characterize patients with metabolic dysfunction according to MAFLD, MASLD, and metabolic syndrome criteria (i.e., the population of interest regarding SLD) and validate the relevance of steatosis for cardiovascular health in these patients.

## 2 Materials and methods

### 2.1 Patients

A total of 4,286 asymptomatic adults participating in the SAKKOPI-study aged 45–80 years undergoing screening colonoscopy were included (11). In brief, the SAKKOPI study included asymptomatic adults participating in opportunistic screening for colorectal cancer. Of note, none of the included individuals had been diagnosed or suspected of having liver disease (e.g., viral hepatitis and cholestatic or autoimmune liver disease). Patients with established CVD or incomplete information on any component of metabolic dysfunction, as detailed below, were excluded.

### 2.2 Definitions

Hepatic steatosis was diagnosed using abdominal ultrasound representing the currently recommended methods for diagnosis of steatosis in the general population (2, 12). Specifically, steatosis was diagnosed if areas of significant increased echogenicity in relation to the renal parenchyma were found.

MASLD was diagnosed according to the recent expert consensus (2) if steatosis was accompanied by one of the following criteria of metabolic dysfunction: (I) overweight (BMI ≥ 25 kg/m²) or waist circumference ≥94/80 cm in Caucasian men and women, (II) blood pressure ≥130/85 mmHg or specific drug treatment, (III) triglycerides ≥150 mg/dL or specific drug treatment, (IV) plasma HDL-cholesterol <40 mg/dL for men and <50 mg/dL for women or specific drug treatment, (V) type 2 diabetes or prediabetes (i.e., fasting blood glucose 100 mg/dL to 125 mg/dL, or 2h post-load glucose levels 140 mg/dL to 199 mg/dL or HbA1c 5.7%–6.4%). MAFLD was diagnosed as proposed in 2020 (1) in the presence of hepatic steatosis and either T2DM, overweight (BMI ≥ 25 kg/m²) or BMI < 25 kg/m² with ≥2 metabolic abnormalities: (I) waist circumference ≥102/88 cm in Caucasian men and women, (II) blood pressure ≥130/85 mmHg or specific drug treatment, (III) triglycerides ≥150 mg/dL or specific drug treatment, (IV) plasma HDL-cholesterol <40 mg/dL for men and <50 mg/dL for women or specific drug treatment, (V) prediabetes (i.e., fasting blood glucose 100 mg/dL to 125 mg/dL, or 2h post-load glucose levels 140 mg/dL to 199 mg/dL or HbA1c 5.7% to 6.4%), (VI) homeostasis model assessment-insulin resistance score (HOMA-IR) ≥2.5, or (VII) plasma C-reactive protein (CRP) level >0.5 mg/dL. The metabolic syndrome was diagnosed according to the IDF/AHA/NHLBI consensus definition (13). Insulin resistance was assessed using HOMA-IR ≥2.5, 10-year cardiovascular risk was assessed using the recently updated SCORE2 and ASCVD risk scores from the European and American cardiology societies. Serum ferritin, CRP, and uric acid were assessed as surrogate markers of subclinical (metabolic) inflammation (14, 15). Alcohol consumption was calculated as standard drinks/week according to a detailed food frequency questionnaire (16).

### 2.3 Statistics

Statistical analyses were performed using R 4.3.1 (R Core Team, R Foundation for Statistical Computing, Vienna, Austria). Continuous variables were presented as mean ± standard deviation or median [interquartile range (IQR)], while categorical variables were reported as the number (proportion) of patients with/without a certain characteristic. The IQR is given as the range between the 25th and 75th percentiles. Student’s t-test was used for group comparisons of normally distributed variables and the Kruskal–Wallis test for non-normally distributed variables, respectively. Group comparisons of categorical variables were performed using Fisher’s exact test or Pearson’s Chi-squared, as appropriate. Multivariable linear regression analysis was used to identify factors associated with cardiovascular risk (dependent variable: SCORE2/ASCVD), and multivariable binary logistic regression analysis for factors associated with hepatic steatosis (dependent variable: steatosis yes/no). The importance for explaining the variance in hepatic steatosis was assessed using the Akaike information criterion applying forward selection of independent variables. Regression analyses were performed in the following subgroups: overall cohort, metabolic dysfunction according to MAFLD criteria, metabolic dysfunction according to MASLD criteria, and metabolic dysfunction according to metabolic syndrome criteria. *p* < 0.05 was considered statistically significant.

## 3 Results

### 3.1 Prevalence of hepatic steatosis and metabolic dysfunction

The mean age was 59.4 ± 8.5 years, with 2212 (51.6%) males and a mean BMI was 27.0 ± 4.5 kg/m², 960 (22.4%) were obese (BMI ≥ 30 kg/m²), 396 (9.2%) had type 2 diabetes mellitus (**Table 1**). In total, 1,903 individuals (44.4%) were diagnosed with hepatic steatosis. They were characterized by more profound metabolic dysfunction including insulin resistance [896 (47.1%) vs. 290 (12.2%), *p* < 0.001] and central obesity [waist circumference ≥102/88 cm, 1,366 (71.8%) vs. 883 (37.1%), *p* < 0.001] among others. This resulted in a greater incidence of (subclinical) inflammation indicated by higher median CRP (0.23 mg/dL vs. 0.13 mg/dL), ferritin (153 mg/dL vs. 95 mg/dL) and uric acid (6.3 mg/dL vs. 5.2 mg/dL, all *p* < 0.001). In line, 10-year cardiovascular risk was higher as assessed by SCORE2 (7.8 points vs. 5.1 points, *p* < 0.001) and ASCVD (17.9 points vs. 10.8 points, *p* < 0.001).

**Table 1:** Patient characteristics of the overall cohort and compared between patients with and without hepatic steatosis.

Patient characteristics	Overall cohort *n* = 4286	Hepatic steatosis *n* = 1903 (44.4%)	No hepatic steatosis *n* = 2383 (55.6%)	*P*-value
Age, years	59.4 ± 8.5	58.8 ± 8.6	60.0 ± 8.2	**< 0.001**
Female sex	2074 (48.4%)	698 (36.7%)	1376 (57.7%)	**< 0.001**
Metabolic syndrome	1434 (33.5%)	1020 (53.6%)	414 (17.4%)	**< 0.001**
T2DM	396 (9.2%)	282 (14.8%)	114 (4.8%)	**< 0.001**
Prediabetes*	2110 (49.2%)	1157 (60.8%)	953 (40.0%)	**< 0.001**
HOMA-IR	1.70 [1.15–2.65]	2.39 [1.65–3.54]	1.33 [0.944–1.87]	**< 0.001**
HOMA-IR ≥ 2.5	1186 (27.7%)	896 (47.1%)	290 (12.2%)	**< 0.001**
Arterial hypertension*	2968 (69.2%)	1495 (78.6%)	1473 (61.8%)	**< 0.001**
BMI, kg/m²	27.0 ± 4.5	29.4 ± 4.5	25.1 ± 3.5	**< 0.001**
Obesity	960 (22.4%)	758 (39.8%)	202 (8.5%)	**< 0.001**
Waist circumference, cm	96.0 [88.0–105]	103 [96.0–110]	91.0 [83.0–98.0]	**< 0.001**
Waist circumference ≥102/88cm	2249 (52.5%)	1366 (71.8%)	883 (37.1%)	**< 0.001**
WHR	0.94 [0.88–0.99]	0.97 [0.92–1.0]	0.91 [0.86–0.96]	**< 0.001**
Hypertrigyleridemia*	1029 (24.0%)	684 (35.9%)	345 (14.5%)	**< 0.001**
Low HDL*	649 (15.1%)	426 (22.4%)	223 (9.4%)	**< 0.001**
Uric acid, mg/dL	5.60 [4.70–6.70]	6.30 [5.30–7.20]	5.20 [4.30–6.10]	**< 0.001**
CRP, mg/dL	0.18 [0.10–0.36]	0.23 [0.12–0.44]	0.13 [0.08–0.27]	**< 0.001**
Ferritin, ng/mL	118 [65–203]	153 [90–258]	95 [53–159]	**< 0.001**
SCORE2	6.2 [3.6–10.2]	7.8 [4.8–11.8]	5.1 [2.9–8.7]	**< 0.001**
ASCVD	13.6 [7.7–23.2]	17.9 [10.7–28.2]	10.8 [6.1–18.6]	**< 0.001**
FIB-4	1.14 [0.90–1.47]	1.14 [0.89–1.48]	1.13 [0.91–1.46]	0.934
Alcohol – tea totalers	1496 (34.9%)	606 (31.8%)	890 (37.3%)	**< 0.001**
Alcohol – < 1 drink/day	1686 (39.3%)	711 (37.4%)	975 (40.9%)	
Alcohol – < 2/3 drinks/day	751 (17.5%)	388 (20.4%)	363 (15.2%)	
Alcohol – abusers	102 (2.4%)	72 (3.8%)	30 (1.3%)	

BMI, body mass index; CRP, C-reactive protein; FIB-4 reactive protein fibrosis 4 score; HOMA-IR, homeostasis model assessment-insulin resistance score; T2DM, type 2 diabetes mellitus; WHR, waist hip ratio; *as defined by the MAFLD, MASLD, and metabolic syndrom criteria.

Bold P values indicate those who meet the criterion for statistical significance (<0.05).

### 3.2 Comparison of NAFLD, MAFLD, MASLD, and metabolic syndrome criteria

**Figure 1:**
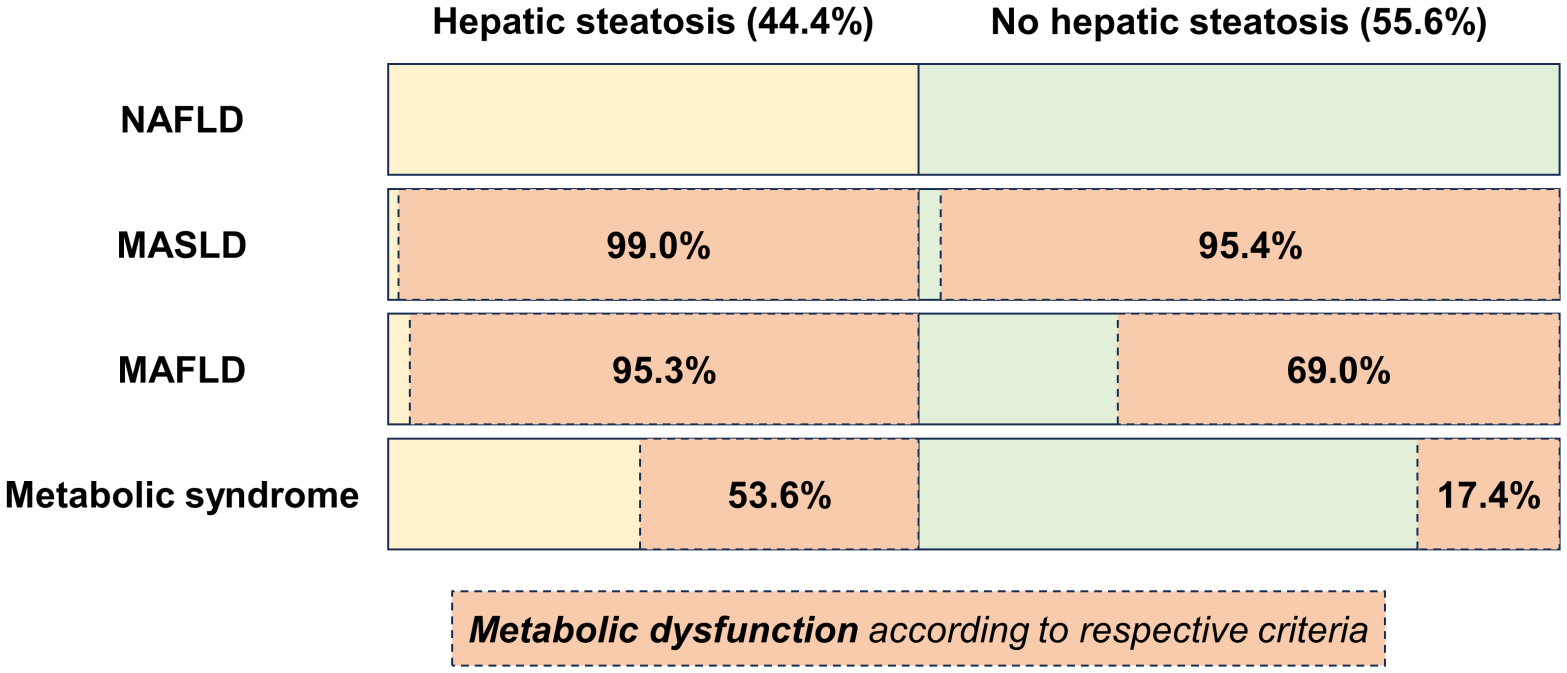
Distribution of hepatic steatosis and prevalence of metabolic dysfunction according to different definitions for MASLD, MAFLD, and metabolic syndrome, respectively.

Importantly, from 1,903 subjects with hepatic steatosis, 1,884 individuals (99.0% of the patients with hepatic steatosis) met the MASLD definition, 1,814 (95.3%) met the MAFLD definition. Additionally, 1,020 individuals (53.6%) diagnosed with hepatic steatosis fulfilled the definition of metabolic syndrome (**Figure 1**). In contrast, 2,273 (95.4%) individuals without steatosis also met MASLD criteria for metabolic dysfunction compared to 1,644 (69.0%) that met the MAFLD criteria and 414 (17.4%) that met the criteria for metabolic syndrome.

### 3.3 Factors associated with hepatic steatosis

Next, we applied a stepwise approach to identify factors explaining the presence of hepatic steatosis (**Table 2**). We assessed these factors in the overall cohort (i.e., NAFLD approach) and in patients with metabolic dysfunction according to MASLD criteria, MAFLD criteria, and patients with metabolic syndrome. Interestingly, waist circumference, insulin resistance (as assessed by HOMA-IR), and triglycerides ranked among the five most relevant variables for the differentiation between patients with and without hepatic steatosis across all subgroups. In contrast, age, sex and other covariables only provided little additional information. Full models can be found as **Supplementary Material Tables S1–S4**.

**Table 2:** Overview of the five most relevant factors explaining the presence of hepatic steatosis in the overall cohort (i.e., NAFLD approach) and subgroups with metabolic dysfunction according to the MASLD, MAFLD, and metabolic syndrome criteria.

	Relevance for explaining the presence of steatosis			
Dependent variable: Hepatic steatosis	Overall cohort (NAFLD)	Metabolic dysfunction according to…		
		MASLD	MAFLD	Metabolic syndrome
Waist circumference, per cm	1	1	1	1
HOMA-IR*, per log	2	2	2	3
ALT, per U/L	3	3	3	**-**
Triglycerides*, per log	4	4	4	2
BMI, per kg/m²	5	5	5	**-**
Systolic BP, per mmHg	–	–	–	4
Glucose, per mg/dL	–	–	–	5

A stepwise approach adding covariables according to their explanatory relevance (as assessed by the Akaike information criterion) was applied starting with the following parameters: age, sex, BMI, HOMA-IR, WC, ferritin, CRP, GGT, HDL, LDL, cholesterol, triglycerides, uric acid, alcohol, systolic blood pressure, ALT, AST, fasting blood glucose, OGTT, and TSH. Full models as well as regression coefficients can be found in the supplement.

*These parameters were log-transformed for regression analyses; BMI, body mass index; HOMA-IR, homeostasis model assessment of insulin.

### 3.4 Factors associated with cardiovascular risk

To test whether hepatic steatosis was independently linked to elevated cardiovascular risk in the overall cohort and subjects with metabolic dysfunction according to different definitions, we performed multivariable linear regression analyses correcting for age, sex, waist circumference, HOMA-IR, and triglycerides. Importantly, hepatic steatosis did not show an independent association with SCORE2 (**Table 3**) or ASCVD (**Supplementary Material Table S5**) in the overall cohort or any of the subgroups with increasing severity of metabolic syndrome, while significant and robust associations were observed for age, sex, waist circumference, insulin resistance, and triglycerides.

**Table 3:** Linear regression analysis investigating factors associated with cardiovascular risk as assessed by SCORE2 in the overall cohort (**A**, i.e., NAFLD-approach), and in subgroups with metabolic dysfunction according to the MASLD criteria (**B**), MAFLD criteria (**C**), or metabolic syndrome criteria (**D**).

Dependent variable: SCORE2	Factors associated with cardiovascular risk		
A – Overall cohort	Adjusted B	95% CI	*P*-value
Age, per year	0.442	0.429–0.455	**< 0.001**
Female sex	−2.972	−3.203–(−2.740)	**< 0.001**
Hepatic steatosis	0.060	−0.193–0.314	0.642
Waist circumference, per cm	0.021	0.010–0.032	**< 0.001**
HOMA-IR*, per log	1.051	0.772–1.331	**< 0.001**
Triglycerides*, per log	1.707	1.464–1.950	**< 0.001**
B – MASLD			
Age, per year	0.442	0.429–0.455	**< 0.001**
Female sex	−3.053	−3.291–(−2.814)	**< 0.001**
Hepatic steatosis	0.048	−0.211–0.306	0.717
Waist circumference, per cm	0.019	0.008–0.030	**< 0.001**
HOMA-IR*, per log	1.058	0.775–1.342	**< 0.001**
Triglycerides*, per log	1.675	1.428–1.922	**< 0.001**
C – MAFLD			
Age, per year	0.454	0.439–0.469	**< 0.001**
Female sex	−3.171	−3.44–(−2.902)	**< 0.001**
Hepatic steatosis	0.978	−0.188–0.384	0.503
Waist circumference, per cm	0.019	0.006–0.032	**0.005**
HOMA-IR*, per log	1.046	0.736–1.356	**< 0.001**
Triglycerides*, per log	1.813	1.536–2.090	**< 0.001**
D – Metabolic syndrome			
Age, per year	0.470	0.442–0.497	**< 0.001**
Female sex	−3.677	−4.184–(-3.170)	**< 0.001**
Hepatic steatosis	−0.244	−0.787–0.299	0.378
Waist circumference, per cm	0.030	0.006–0.054	**0.015**
HOMA-IR*, per log	1.346	0.834–1.858	**< 0.001**
Triglycerides*, per log	1.959	1.479–2.439	**< 0.001**

Age, sex, hepatic steatosis, and the three most relevant variables to explain the presence of hepatic steatosis in this cohort (i.e., waist circumference, HOMA-IR assessing insulin resistance, and triglycerides) were used as covariables.

*These parameters were log-transformed for regression analyses; HOMA-IR, homeostasis model assessment of insulin.

Bold P values indicate those who meet the criterion for statistical significance (<0.05).

## 4 Discussion

In recent years, the hepatology community has introduced the term MAFLD (2020) (1) and MASLD (2023) (2) together with an updated definition requiring the presence of metabolic dysfunction to be met. Although these criteria aim to focus on patients with increased metabolic dysregulation and therefore likely progressive disease, it is currently unclear whether this approach effectively identifies patients with hepatic steatosis and worse prognosis, particularly regarding CVD (17). Importantly, these criteria also differ between each other, with the recent MASLD criteria being very broad and met not only by 99.0% of patients with steatosis but also by 95.4% of patients without. Although they are highly inclusive, they lack granularity since almost all of our cohort, comprising individuals aged 45–80 years (i.e., the target population for MASLD prevention strategies) meet them. Although previous MAFLD criteria were more stringent, applying the established criteria for metabolic syndrome identifies the subgroup of patients with steatosis and profound metabolic dysfunction that are barely found in subjects without steatosis (17.4%).

In summary, our findings raise doubts about whether hepatic steatosis is truly an independent risk factor for CVD if proper adjustment for disease-driving factors (especially insulin resistance and central obesity, among others) is performed (6, 7). Here, it remains uncertain whether the current definition of “metabolic dysfunction” necessary for diagnosing MASLD is precise enough to identify the target population for intensified screening or treatment. However, the presence of hepatic steatosis in general indicates a deeper metabolic imbalance.

Importantly, three parameters that are rarely adjusted for in large population-based studies and meta-analyses explain the greatest amount of variance between individuals with and without steatosis: waist circumference indicating visceral fat mass, HOMA-IR corresponding to insulin resistance, and triglycerides.

Next, while male sex was clearly associated with the presence of steatosis in univariable analysis as well as the literature, we were able to demonstrate that metabolic comorbidities could explain these differences across sexes while we could not identify an independent association of sex with hepatic steatosis. Therefore, women are not “protected” from steatosis but rather have a healthier body composition, lifestyle, and metabolic parameters, which are often insufficiently accounted for in population-based cohorts.

Finally, hepatic steatosis was not independently associated with cardiovascular risk in the presence or absence of metabolic dysfunction. Conversely, the aforementioned metabolic comorbidities were the main drivers behind this association. Clear associations of insulin resistance (18, 19) and visceral obesity (20, 21) with cardiovascular mortality require adjusting for these factors when proposing an independent relationship of steatosis with cardiovascular risk to avoid “omitted-variables” bias.

This study has several limitations. Most importantly, hepatic steatosis was diagnosed using ultrasound with limitations in detecting <5% of hepatic steatosis, yet being the currently recommended tool to screen for and/or diagnose steatosis by the European Association for the Study of the Liver (EASL) (22) and current MASLD consensus statement endorsed by all major international and national societies (2). Second, the cross-sectional design is a limitation as data on cardiovascular events during follow-up were not systematically assessed and could therefore not be analyzed. However, SCORE2 (and ASCVD) were used as the most precise surrogate for cardiovascular risk currently endorsed by the European Society of Cardiology as an updated score to estimate 10-year fatal and non-fatal CVD risk in patients without previous CVD aged 40–69 years in Europe (23).

In conclusion, we confirm that visceral obesity and insulin resistance are driving factors in the development of hepatic steatosis, even when metabolic dysfunction is present. Most importantly, these factors rather than hepatic steatosis *per se* explain cardiovascular. Thus, they should be taken into account when claiming independent associations with cardiovascular risk.

## Data availability statement

The datasets presented in this article are not readily available because of European data security regulations. Requests to access the datasets should be directed to Christian Datz; c.datz@kh-oberndorf.at.

## Ethics statement

The studies involving humans were approved by Ethikkommission für das Bundesland Salzburg. Sebastian-Stief-Gasse 2 5020 Salzburg. The studies were conducted in accordance with the local legislation and institutional requirements. The participants provided their written informed consent to participate in this study.

## Author contributions

Conception and design: GS, LB, DN, CD; Administrative support: GS, LB, DN, CD; Provision of study materials or patients: all authors; Collection and assembly of data: all authors; Data analysis and interpretation: GS, LB, DN, CD; Manuscript writing: GS, LB, DN, CD; Final approval of manuscript: All authors.
