# Supplementary material for: Insulin resistance and central obesity determine hepatic steatosis and explain cardiovascular risk in steatotic liver disease
Source: Front Endocrinol (Lausanne). 2023 Sep 29;14:1244405. doi: 10.3389/fendo.2023.1244405 (PMC10570507; doi:10.3389/fendo.2023.1244405)
Supplement: Supplementary file 1 [file DataSheet_1.docx]

**SUPPLEMENT**

**Supplementary table 1**. Full binary logistic regression analysis investigating factors associated with hepatic steatosis in the overall cohort. A stepwise approach adding covariables according to their explanatory relevance (as assessed by the Akaike information criterion) was applied starting with the following parameters: Age, sex, BMI, HOMA-IR, WC, ferritin, CRP, GGT, HDL, LDL, cholesterol, triglycerides, uric acid, alcohol, systolic blood pressure, ALT, AST, fasting blood glucose, OGTT and TSH.

|  | **Factors associated with hepatic steatosis** | | | |
| --- | --- | --- | --- | --- |
| Dependent variable: Hepatic steatosis | Relevance for explaining presence of steatosis | Adjusted OR | 95%CI | P value |
| WC, per cm | 1 | 1.035 | 1.021-1.049 | **<0.001** |
| HOMA-IR*, per log | 2 | 3.117 | 2.345-4.165 | **<0.001** |
| ALT, per U/L | 3 | 1.027 | 1.017-1.038 | **<0.001** |
| Triglycerides*, per log | 4 | 1.519 | 1.241-1.862 | **<0.001** |
| BMI, per kg/m² | 5 | 1.120 | 1.080-1.162 | **<0.001** |
| Alcohol – <1 drink/day | 6 | 1.159 | 0.954-1.409 | 0.137 |
| Alcohol – <2/3 drinks/day |  | 1.410 | 1.101-1.806 | **0.006** |
| Alcohol – abusers |  | 3.112 | 1.715-5.805 | **<0001** |
| OGTT, per mg/dL | 7 | 1.005 | 1.002-1.007 | **<0.001** |
| Ferritin*, per log | 8 | 1.177 | 1.054-1.315 | **0.004** |
| GGT*, per log | 9 | 1.268 | 1.073-1.498 | **0.005** |
| Uric acid, per mg/dL | 10 | 1.090 | 1.017-1.167 | **0.014** |
| Systolic BP, per mmHg | 11 | 1.005 | 1.001-1.010 | **0.030** |
| AST, per U/L | 12 | 0.986 | 0.972-1.000 | **0.036** |
| *these parameters were log-transformed for regression analyses; BMI – body mass index; HOMA-IR - homeostasis model assessment of insulin; OGTT – oral glucose tolerance test after 2h; OR – odds ratio; WC - waist circumference; | | | | |

**Supplementary table 2**. Full binary logistic regression analysis investigating factors associated with hepatic steatosis in patients meeting the definition of metabolic dysfunction according to the MASLD criteria. A stepwise approach adding covariables according to their explanatory relevance (as assessed by the Akaike information criterion) was applied starting with the following parameters: Age, sex, BMI, HOMA-IR, WC, ferritin, CRP, GGT, HDL, LDL, cholesterol, triglycerides, uric acid, alcohol, systolic blood pressure, ALT, AST, fasting blood glucose, OGTT and TSH.

|  | **Factors associated with hepatic steatosis** | | | |
| --- | --- | --- | --- | --- |
| Dependent variable: Hepatic steatosis | Relevance for explaining presence of steatosis | Adjusted OR | 95%CI | P value |
| WC, per cm | 1 | 1.041 | 1.026-1.056 | **<0.001** |
| HOMA-IR*, per log | 2 | 3.025 | 2.2724.046 | **<0.001** |
| ALT, per U/L | 3 | 1.028 | 1.018-1.039 | **<0.001** |
| Triglycerides*, per log | 4 | 1.579 | 1.288-1.939 | **<0.001** |
| BMI, per kg/m² | 5 | 1.112 | 1.070-1.156 | **<0.001** |
| Alcohol – <1 drink/day | 6 | 1.184 | 0.970-1.445 | 0.096 |
| Alcohol – <2/3 drinks/day |  | 1.509 | 1.168-1.951 | **0.002** |
| Alcohol – abusers |  | 3.316 | 1.816-6.224 | **<0.001** |
| OGTT, per mg/dL | 7 | 1.004 | 1.002-1.007 | **<0.001** |
| Ferritin*, per log | 8 | 1.214 | 1.084-1.362 | **<0.001** |
| GGT*, per log | 9 | 1.263 | 1.067-1.494 | **0.007** |
| Systolic BP, per mmHg | 11 | 1.007 | 1.002-1.012 | **0.009** |
| Uric acid, per mg/dL | 10 | 1.108 | 1.029-1.194 | **0.007** |
| AST, per U/L | 12 | 0.984 | 0.971-0.998 | **0.023** |
| Female sex | 13 | 1.267 | 1.006-1.598 | **0.046** |
| *these parameters were log-transformed for regression analyses; BMI – body mass index; HOMA-IR - homeostasis model assessment of insulin; OGTT – oral glucose tolerance test after 2h; OR – odds ratio; WC - waist circumference; | | | | |

**Supplementary table 3**. Full binary logistic regression analysis investigating factors associated with hepatic steatosis in patients meeting the definition of metabolic dysfunction according to the MAFLD criteria. A stepwise approach adding covariables according to their explanatory relevance (as assessed by the Akaike information criterion) was applied starting with the following parameters: Age, sex, BMI, HOMA-IR, WC, ferritin, CRP, GGT, HDL, LDL, cholesterol, triglycerides, uric acid, alcohol, systolic blood pressure, ALT, AST, fasting blood glucose, OGTT and TSH.

|  | **Factors associated with hepatic steatosis** | | | |
| --- | --- | --- | --- | --- |
| Dependent variable: Hepatic steatosis | Relevance for explaining presence of steatosis | Adjusted OR | 95%CI | P value |
| WC, per cm | 1 | 1.031 | 1.017-1.046 | **<0.001** |
| HOMA-IR*, per log | 2 | 3.139 | 2.336-4.244 | **<0.001** |
| ALT, per U/L | 3 | 1.027 | 1.016-1.038 | **<0.001** |
| Triglycerides*, per log | 4 | 1.408 | 1.220-1.863 | **<0.001** |
| BMI, per kg/m² | 5 | 1.119 | 1.078-1.164 | **<0.001** |
| Alcohol – <1 drink/day | 6 | 1.112 | 0.905-1.367 | 0.331 |
| Alcohol – <2/3 drinks/day |  | 1.319 | 1.016-1.713 | **0.037** |
| Alcohol – abusers |  | 3.738 | 1.937-7.606 | **<0.001** |
| Ferritin*, per log | 7 | 1.200 | 1.068-1.349 | **0.002** |
| OGTT, per mg/dL | 8 | 1.004 | 1.002-1.007 | **<0.001** |
| GGT*, per log | 9 | 1.252 | 1.049-1.495 | **0.013** |
| Uric acid, per mg/dL | 10 | 1.093 | 1.016-1.175 | **0.017** |
| Systolic BP, per mmHg | 11 | 1.006 | 1.001-1.011 | **0.020** |
| AST, per U/L | 12 | 0.984 | 0.971-0.999 | **0.030** |
| *these parameters were log-transformed for regression analyses; BMI – body mass index; HOMA-IR - homeostasis model assessment of insulin; OGTT – oral glucose tolerance test after 2h; OR – odds ratio; WC - waist circumference; | | | | |

**Supplementary table 4**. Full binary logistic regression analysis investigating factors associated with hepatic steatosis in patients meeting the definition of metabolic dysfunction according to the metabolic syndrome criteria. A stepwise approach adding covariables according to their explanatory relevance (as assessed by the Akaike information criterion) was applied starting with the following parameters: Age, sex, BMI, HOMA-IR, WC, ferritin, CRP, GGT, HDL, LDL, cholesterol, triglycerides, uric acid, alcohol, systolic blood pressure, ALT, AST, fasting blood glucose, OGTT and TSH.

|  | **Factors associated with hepatic steatosis** | | | |
| --- | --- | --- | --- | --- |
| Dependent variable: Hepatic steatosis | Relevance for explaining presence of steatosis | Adjusted OR | 95%CI | P value |
| WC, per cm | 1 | 1.082 | 1.062-1.103 | **<0.001** |
| Triglycerides*, per log | 2 | 5.583 | 4.140-7.583 | **<0.001** |
| HOMA-IR*, per log | 3 | 2.827 | 1.920-4.169 | **<0.001** |
| Systolic BP, per mmHg | 4 | 1.027 | 1.020-1.034 | **<0.001** |
| Glucose, per mg/dL | 5 | 1.026 | 1.014-1.034 | **<0.001** |
| OGTT, per mg/dL | 6 | 1.007 | 1.004-1.010 | **<0.001** |
| BMI, per kg/m² | 7 | 1.067 | 1.017-1.120 | **0.008** |
| HDL, per mg/dL | 8 | 0.964 | 0.953-0.975 | **<0.001** |
| Female sex | 9 | 3.110 | 2.222-4.372 | **<0.001** |
| GGT*, per log | 10 | 1.407 | 1.139-1.740 | **0.002** |
| Ferritin*, per log | 11 | 1.303 | 1.118-1.522 | **<0.001** |
| ALT, per U/L | 12 | 1.017 | 1.006-1.028 | **0.002** |
| AST, per U/L | 13 | 0.983 | 0.968-0.986 | **0.025** |
| Alcohol – <1 drink/day | 14 | 1.021 | 0.774-1.349 | 0.882 |
| Alcohol – <2/3 drinks/day |  | 1.186 | 0.834-1.686 | 0.342 |
| Alcohol – abusers |  | 2.713 | 1.372-5.385 | **0.004** |
| CRP*, per log | 15 | 6.470 | 0.393-1.053 | 0.083 |
| *these parameters were log-transformed for regression analyses; BMI – body mass index; HOMA-IR - homeostasis model assessment of insulin; OGTT – oral glucose tolerance test after 2h; OR – odds ratio; WC - waist circumference; | | | | |

**Supplementary table 5**. Linear regression analysis investigating factors associated with cardiovascular risk as assessed by ASCVD in the overall cohort (**A**, i.e., NAFLD-approach), and in subgroups with metabolic dysfunction according to the MASLD criteria (**B**), MAFLD criteria (**C**) or metabolic syndrome criteria (**D**). Age, sex, hepatic steatosis, and the three most relevant variables to explain the presence of hepatic steatosis in this cohort (i.e., waist circumference, HOMA-IR assessing insulin resistance, and triglycerides) were used as covariables.

| Dependent variable: ASCVD | **Factors associated with cardiovascular risk** | | |
| --- | --- | --- | --- |
| **A – Overall cohort** | Adjusted B | 95%CI | P value |
| Age, per year | 0.834 | 0.795-0.873 | **<0.001** |
| Female sex | -9.519 | -10.204-(-8.834) | **<0.001** |
| Hepatic steatosis | 0.310 | -0.446-1.067 | 0.421 |
| Waist circumference, per cm | 0.062 | 0.293-0.945 | **0.003** |
| HOMA-IR*, per log | 3.185 | 2.335-4.034 | **<0.001** |
| Triglycerides*, per log | 5.778 | 5.050-6.506 | **<0.001** |
| **B - MASLD** | | | |
| Age, per year | 0.833 | 0.793-0.873 | **<0.001** |
| Female sex | -9.875 | -10.583-(-9.166) | **<0.001** |
| Hepatic steatosis | 0.310 | -0.462-1.083 | 0.431 |
| Waist circumference, per cm | 0.054 | 0.020-0.087 | **0.002** |
| HOMA-IR*, per log | 3.149 | 2.286-4.011 | **<0.001** |
| Triglycerides*, per log | 5.645 | 4.902-6.388 | **<0.001** |
| **C - MAFLD** | | | |
| Age, per year | 0.879 | 0.833-0.924 | **<0.001** |
| Female sex | -10.371 | -11.175-(-9.567) | **<0.001** |
| Hepatic steatosis | 0.310 | -0.549-1.170 | 0.479 |
| Waist circumference, per cm | 0.058 | 0.019-0.098 | **0.004** |
| HOMA-IR*, per log | 3.120 | 2.171-4.068 | **<0.001** |
| Triglycerides*, per log | 6.235 | 5.399-7.071 | **<0.001** |
| **D – Metabolic syndrome** | | | |
| Age, per year | 0.978 | 0.898-1.059 | **<0.001** |
| Female sex | -12.696 | -14.157-(-11.236) | **<0.001** |
| Hepatic steatosis | -0.394 | -1.967-1.180 | 0.624 |
| Waist circumference, per cm | 0.081 | 0.010-0.151 | **0.025** |
| HOMA-IR*, per log | 3.836 | 2.341-5.331 | **<0.001** |
| Triglycerides*, per log | 6.534 | 5.121-7.946 | **<0.001** |
| *these parameters were log-transformed for regression analyses; HOMA-IR - homeostasis model assessment of insulin; | | | |
